# Evaluation of Molecular and Cellular Alterations Induced by Neuropathic Pain in Rat Brain Glial cells

**DOI:** 10.22037/ijpr.2020.113052.14089

**Published:** 2021

**Authors:** Mohsen Rezaei, Lida Karimian, Bizhan Shafaghi, Maryam Noubarani, Maryam Salecheh, Mohammad Shafi Dehghani, Mohammad Reza Eskandari, Jalal Pourahmad

**Affiliations:** a *Department of Toxicology, Faculty of Medical Sciences, Tarbiat Modares University, Tehran, Iran. *; b *Department of Pharmacology and Toxicology, Faculty of Pharmacy, Shahid Beheshti University of Medical Sciences, Tehran, Iran. *; c *Department of Pharmacology and Toxicology, School of Pharmacy, Zanjan University of Medical Sciences, Zanjan, Iran. *; d *Department of Pharmacology and Toxicology, School of Pharmacy, Jundishapur University of Medical Sciences, Ahvaz, Iran. *; e *Zanjan Pharmaceutical Nanotechnology Research Center (ZPNRC), Zanjan University of Medical Sciences, Zanjan, Iran. *

**Keywords:** Glial cell, Oxidative stress, Lysosome, Cell death, Proteolysis, Caspase-3

## Abstract

Neuropathic pain originates from illness or damage of the nervous system and affects the somatosensory system. Recently, many efforts have been made to illuminate the influences of neuropathic pain in different parts of central nervous system (CNS). However, the toxic consequences of neuropathic pain in glial cells, which involve in the control of pain is poorly understood. Therefore, the present study aimed to assess the molecular and cellular effects of neuropathic pain in the glial cells of rat brain. Induction of neuropathic pain in rats was associated with oxidative stress as evident by elevated reactive oxygen species (ROS) formation as well as reversible glutathione (GSH) depletion in the glial cells. Moreover, neuropathic pain caused mitochondrial membrane potential collapse (∆Ψm%), lysosomal membrane rapture, and proteolysis, probably due to ROS-induced MPT pore opening. These toxic events could cause cytochrome c release from intermembrane space into the cytosole and trigger caspase activation pathway. Our finding confirmed that the activity of caspase-3 was significantly increased in the glial cells as a core component of the apoptotic machinery. In conclusion, the neuropathic pain induces ROS generation as the major cause of GSH depletion along with mutual mitochondrial/lysosomal potentiation (cross-talk) of oxidative stress in the glial cells. Subsequently, this toxic cross-talk can induce proteolysis and trigger apoptosis by caspase-3 activation in the glial cells of rat brain.

## Introduction

 It is believed that pain is a warning system that plays major role in the human body, including warning about damaged tissues, taking away from the source of the damage, and ultimately reduced activity to accelerate the healing process (1-3). Pain can be psychogenic or somatogenic, in which the somatogenic pain comprises nociceptive and neuropathic pain. Somatic or visceral nerve fibers activation by harmful agents create the nociceptive pain, whereas dysfunction of the central or peripheral nervous system leads to neuropathic pain (4, 5). The neuropathic pain, which can be due to peripheral or central neuropathy, is common and seriously disables millions of people around the world. The peripheral neuropathy arises from trauma, muscle atrophy along the nerve, connective tissue disease, malignancies, and diabetes, while central neuropathy occurs due to overlap disks, sub arachnoids inflammation, *etc.* (6, 7).

Damage to the nervous system has many molecular and cellular consequences and causes transformation and regeneration of neurons in anatomically different parts of nervous system (8-10). Glial cells or simply glia is known as a potent modulator of pain sensation and considered as a tool, which provides a new target for discovery of more effective drugs for pain alleviation (11). In the recent years, glial cells were found to modify neuronal function in the biological and pathological conditions (12). Glial deactivation in the brain or the spinal cord reduced pain induced by factors, such as inflammation of the peripheral nerves, spinal nerve transection, and the inflammation of the skin (13-15). On the other hand, glial cells apparently have no effect on the acute pain responses and specifically involve in pathological pains (16-18).

Chronic neuropathic pain and hyperalgesia possibly provoke neuronal cell death in the dorsal horn of the spinal cord, thus enlightening the processes involved in neuronal damage might identify the mechanisms of chronic neuropathic pain (12, 19). However, most of the consequences of neuropathic pain in glial cells have not yet been completely understood. Thus, the present study was designed to evaluate the molecular and cellular alterations induced by neuropathic pain in rat brain glial cells. Moreover, the role of cellular death in these toxic events was investigated.

## Experimental

*Chemicals*

Bovine serum albumin (BSA), collagenase (from Clostridium histolyticum), N-Ethylmaleimide (NEM), O-phthalaldehyde (OPT), and rhodamine 123 were purchased from Sigma-Al- drich Co. (Taufkirchen, Germany). All other chemicals were of the highest commercial grade available.

*Animals*

Male Sprague-Dawley rats weighing 180 to 200 g were housed in ventilated plastic cages over PWI 8-16 hardwood bedding. There were 12 air changes per h, 12 h light photoperiods, an environmental temperature of 21–23 °C, and a relative humidity of 50–60 %. The animals were fed a standard normal chow diet and given tap water ad libitum. Principles of laboratory animal care (NIH publication No. 85-23, revised 1985) were followed. All experiments were conducted according to the ethical standards and protocols approved by the Committee of Animal Experimentation of Shahid Beheshti University of Medical Sciences, Tehran, Iran (protocol approval number: 88/01/94/6555).

*Induction of neuropathic pain*

Under 10% chloral hydrate (3 mL/kg) anesthesia the skin on the lateral surface of the thigh was incised and a section made directly through the biceps femoris muscle exposing the sciatic nerve and its three terminal branches: the sural, common peroneal, and tibial nerves. The Spared Nerve Injury (SNI) procedure comprised an axotomy and ligation of the tibial and common peroneal nerves leaving the sural nerve intact. The common peroneal and the tibial nerves were tight-ligated with 5.0 silk and sectioned distal to the ligation, removing 2–4 mm of the distal nerve stump. Great care was taken to avoid any contact with or stretching of the intact sural nerve. Muscle and skin were closed in two layers. Crush controls (spared nerve crush group) were performed as above, except that the tibial and common peroneal nerves were crushed for 30 s by a pair of small arterial forceps with smooth protective pads over the blades. At the end of this procedure the nerves were completely flattened and transparent. Sham controls involved exposure of the sciatic nerve and its branches without any lesion (20).

*Mechanical hyperalgesia*

After SNI pain evaluation was performed to confirm the occurrence of allodynia or hyperalgesia in the rat model. With the animals on the elevated grid, a pinprick test was performed using a safety pin. The lateral part of the plantar surface of the paw was briefly stimulated at an intensity sufficient to indent but not penetrate the skin (pin-prick test). The duration of paw withdrawal was recorded, with an arbitrary minimal time of 0.5 s (for the brief normal response) and a maximal cut-off of l0 s (21). 

Our results confirmed the induction of hyperalgesia in the neuropathic pain rat model. The withdrawal duration (in seconds) after a pin prick stimulus to the lateral plantar surface of the paw increased significantly after SNI. The SNI group’s elevated response (pin-prick hyperalgesia) persisted for the entire time the animals were monitored (2 weeks) (Data not shown).

*Study design*

The rats were randomly divided into three different groups of 24 rats each. Group 1 (Control) animals fed with a standard diet and served as a normal control without surgery. Group 2 (Sham) rats that the SNI surgery was performed and was exposed to the sciatic nerve and its three terminal branches but without ligated and cutoﬀ the common peroneal and tibial nerves. Group 3 (Case or neuropathic pain model) rats that the SNI surgery was performed along with ligated and cutoﬀ the common peroneal and tibial nerves.

*Glial cells isolation*

On the second, 6^th^, 10^th^, and 14^th^ days after the surgery, the glia cells were taken. For this, brain was removed and placed in PBS buffer, hemispheres were separated and the bean-shaped hippocampi were isolated. Hippocampus was placed in dishes containing PBS, gently minced and pipetting up and down then incubated in a buffer contains trypsin-EDTA for 5 min at 37 °C. Then, it was smoothly shacked and again incubated for additional 5 min. Afterward, DMEM medium was added. The prepared suspension passed through the funnel (70 mesh, 25 micron) by the vacuum pump for elimination of neurons and oligodendrocytes. The cells were suspended at a density of 10^6^ cells/mL in round bottomed ﬂasks rotating in a water bath maintained at 37 °C in Krebs–Henseleit buffer (pH 7.4), supplemented with 12.5 mM HEPES under an atmosphere of 10% O_2_, 85% N2, and 5% CO2. The cell suspensions were poured into round bottom flasks and were placed in a bioreactor for 15 min. Glia cells was placed in 1 mL of each in eppendorf tubes and was centrifuged for 1 min at 1000 rpm (22).

*Glial cell identification*

To confirm that a majority of isolated cells were glial cells, cells were analyzed for the expression of astrocyte marker glial fibrillary acidic protein (GFAP) through antibody staining. GFAP immunostained samples confirms that 90% of cells present are GFAP positive cells (Immunostaining data not shown).

*Determination of reactive oxygen spices (ROS* *(*

To measure the rate of glial cells ROS formation, non-ﬂuorescent dichloroﬂuorescin diacetate (DCFH-DA) was used that hydrolyze to dichloroﬂuorescin (DCFH) (non-fluorescent) inside the cells. DCFH reacted with intracellular ROS and converted to the highly ﬂuorescent dichloroﬂuorescein (DCF) that outflow the cell. For this purpose, 3 ml of cells were centrifuged for 1 min at 1000, then mixed with 3 mL of DCFH diacetate. The suspension was shaken gently and incubated for 10 min at 37 °C. The amount of ROS was measured according to fluorescence intensity by the fluorescence spectrometer (Excitation: 500 nm, Emission: 520 nm). The results were expressed as ﬂuorescent intensity per 10^6^ cells (23).

*Determination of intracellular reduced glutathione (GSH)*

The amounts of reduced and oxidized glutathione were assessed by the spectroﬂuorometric method with a few modifications (24). For the measurement of GSH, 1 mL of the cell suspension was centrifuged (1000 rpm, 1 min), and the supernatant was removed. Trichloroacetic acid (10%) was added to the cell pellet. After centrifugation (11000 rpm, 1 min), 4.5 mL of the incubation buffer (pH 8) was added to the supernatant (0.5 mL) then mixed with 100 µL of OPT (1 mg/mL). Incubation buffer (1.8 mL) was added to 100 µl of diluted supernatant in the previous step and incubated for 15 min at room temperature. The intensity was measured at Excitation: 350 nm and Emission: 420 nm.

*Measurement of extracellular oxidized glutathione (GSSG)*

1ml of the cell suspension centrifuged (1000 rpm, 1 min) and the supernatant was mixed with trichloroacetic acid 10%. After centrifugation (11000 rpm, min 1), 0.5 mL of the supernatant from the previous step mixed with NEM (0.04 M, 200 µM) for 30 min at room temperature. Measurement was similar to the GSH determination, except for incubation buffer that was replaced by NaOH (0.1 N) instead as diluents. The amount of GSH and GSSG was calculated by calibration curves (24).

*Determination of glutathione precursors for compensation biosynthesis*

After the incubation of glial cells with DMEM medium, the flasks were centrifuged (1000 rpm, 1 min). Incubation buffer (10 mL) was added to the cell pellet and the cell suspension again was transferred into the flask, then incubated with two glutathione precursors, methionine and betaine, for 3 h. The level of GSH was measured by the method described in the previous section.

*Mitochondrial membrane potential collapse (∆* *Ψm* *%) measurement*

For the determination of mitochondrial membrane potential decline, cationic flourogenic probe Rhodamine 123 was used (25). Cell suspension (0.5 mL) was incubated and centrifuged for 1 min at 1000 rpm. The supernatant was removed and Rhodamine (1.5 µM) incubated for 10 min at 37 °C. Then, fluorescence intensity was determined at Excitation: 490 nm and Emission: 520 nm. The capacity of mitochondria to take up the Rhodamine 123 was calculated as the fluorescence difference between the control and test cells.

*Measurement of Caspase-3 Activity*

Caspase-3 activity was assessed in glial cells lysate by ‘‘Sigma’s caspase-3 assay kit (CASP-3-C)’’ (26). This method is based on the hydrolysis of a peptide substrate (AC-DEVD-pNA) by caspase-3 that leads to release the para-nitro aniline (pNA) moiety. The concentration of pNA released from the substrate was determined by absorption at 405 nm (27).

*Assessment of lysosomal membrane stability*

Lysosomal membrane stability was measured using acridine orange dye (28). In this method, 1 ml of cell suspension was mixed with 2 mL acridine orange (5 µg/mL) and incubated for 10 min at 37 °C. Then, the fluorescence intensity from distribution of acridine orange to the cytosol was determined at wavelength Excitation: 495 nm: and Emission: 530 nm.

*Determination of proteolysis*

Proteolysis was measured by fluorescence intensity from tyrosine released into the extracellular space (29). Each of the samples was added trichloroacetic acid (20%) and incubated for 12 h at 4 °C, then, centrifuged (13250 rpm, 10 min). 1-Nitroso-2-naphthol (1 mL) and 1 mL of nitrite acid (10 mg/mL NaNo_2_ in HNO_3_ 20%) were added to the supernatant and incubated for 30 min at 37 °C. After the addition of ethylene chloride, the suspension was shaken strongly, and again centrifuged (13250 rpm, 10 min). The fluorescence intensity was measured at Excitation: 460 nm and Emission: 570 nm. The amount of tyrosine was calculated by tyrosine calibration curve (0-100 µM).

*Statistical analysis*

The homogeneity of variances was tested using Levene’s test. Results were expressed as mean ± SD. All data were statistically analyzed by two-way ANOVA followed by Bonferroni *post hoc* test using GraphPad Prism 8.0. The results with level of significance (*P* < 0.05) were regarded as significant.

## Results

*Effects of neuropathic pain on the markers of oxidative stress*

**Figure 1:**
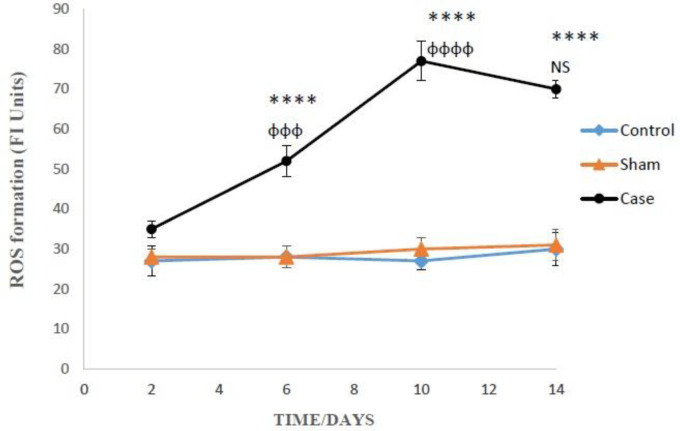
The amounts of ROS production in the glial cells after the induction of neuropathic pain. ROS formation was expressed as fluorescent intensity (FI) units. Values are presented as mean ± SD. ^****^P < 0.0001 Signiﬁcant difference between case and sham groups in corresponding time; ^ɸɸɸ^*P* < 0.001, ^ɸɸɸɸ^*P* < 0.0001 Compared with previous time in case group; NS: Not Significant

ROS formation in the glial cells of pain group was significantly higher than the sham group after 6, 10, and 14 days (*P* < 0.0001), but there was not any significant differences between sham and control groups after induction of neuropathic pain (Figure 1). Bonferroni *post hoc *test also indicated that ROS generation in the glial cells of pain group was time-dependent (*P* < 0.0001).

**Figure 2:**
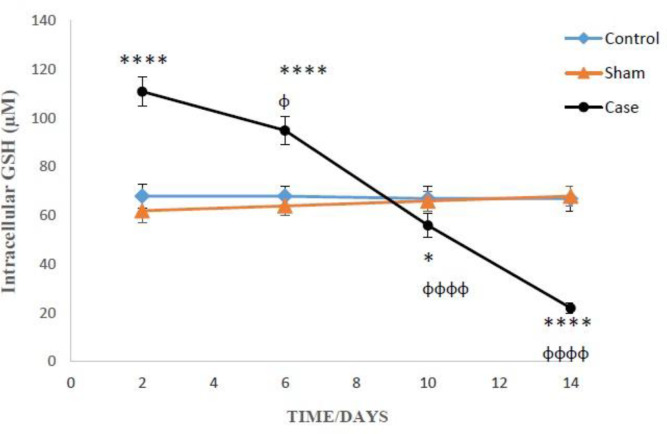
The levels of intracellular GSH the glial cells after the induction of neuropathic pain. Values are presented as mean ± SD. ^*^P < 0.05, ^****^P <0.0001 Signiﬁcant difference between case and sham groups in corresponding time; ^ɸ^*P* < 0.05, ^ɸɸɸɸ^*P* < 0.0001 Compared with previous time in case group

**Figure 3:**
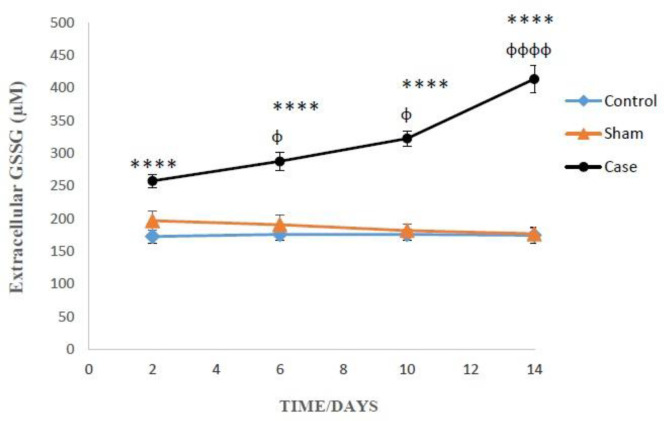
The levels of extracellular GSSG in the glial cells after the induction of neuropathic pain. Values are presented as mean ± SD. ^****^P < 0.0001 Signiﬁcant difference between case and sham groups in corresponding time; ^ɸ^*P* < 0.05, ^ɸɸɸɸ^*P* < 0.0001 Compared with previous time in case group

Figures 2 and 3 present the concentration of reduced and oxidized glutathione, respectively inside and outside the glial cells, at various days after induction of neuropathic pain. The level of intracellular GSH in the pain group on the 2^nd^ and 6^th^ days was considerably higher in contrast the sham group (*P* < 0.0001). On the other hand, GSH in the pain group on the 10^th^ and 14^th^ days were statically lower as compared to the sham group (Figure 2). Besides, two-way ANOVA analysis showed that GSH concentration changes in the glial cells of pain group were time-dependent (*P* < 0.0001). 

As presented in Figure 3, on the 2^nd^, 6^th^, 10^th^ and 14^th^ days after induction of neuropathic pain, the levels of extracellular GSSG significantly raised in the glial cells of pain group in comparison with sham groups (*P* < 0.0001). In addition, Bonferroni *post hoc* test confirmed that GSSG concentration changes in the glial cells of pain group were time-dependent (*P* < 0.0001). 

On the 14^th^ day of pain induction, the levels of GSH in the glial cells of rat neuropathic pain model that incubated with methionine and betaine, the precursors of GSH, were significantly higher than those of the precursors-free pain group (Table 1). The evaluation of glutathione compensatory biosynthesis after incubation of glial cells with methionine and betaine implies that the biosynthesis pathway of glutathione is functionally active. Besides, the concentrations of intracellular GSH and extracellular GSSG in the sham and control groups did not show significant alterations in different time points after the induction of neuropathic pain (Figures 2 and 3). 

*Effects of neuropathic pain on mitochondrial membrane potential collapse*

**Figure 4:**
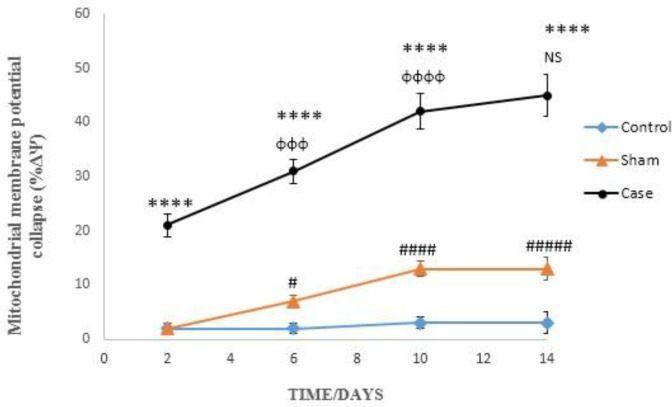
Mitochondrial membrane potential collapse (ΔΨm%) in the glial cells after the induction of neuropathic pain. Values are presented as mean ± SD. ^****^P < 0.0001 Signiﬁcant difference between case and sham groups in corresponding time; ^ɸɸɸ^*P* < 0.001, ^ɸɸɸɸ^*P* < 0.0001 Compared with previous time in case group; NS: Not Significant; ^#^*P *< 0.05, ^#####^*P *< 0.0001 Signiﬁcant difference between sham and control groups in corresponding time

Induction of neuropathic pain caused a significant decline of mitochondrial membrane potential (∆Ψm%) in the glial cells in different time points (*P* < 0.0001). ∆Ψm% in the sham group was significantly higher than control rats at 6, 10, and 14 days after the pain induction (Figure 4). Moreover, two-way ANOVA analysis showed that ΔΨm% in the glial cells of pain group was time-dependent (*P* < 0.0001) as well as in the glial cells of sham group (*P* < 0.05).

*Effects of neuropathic pain on caspase-3 activation*

**Figure 5:**
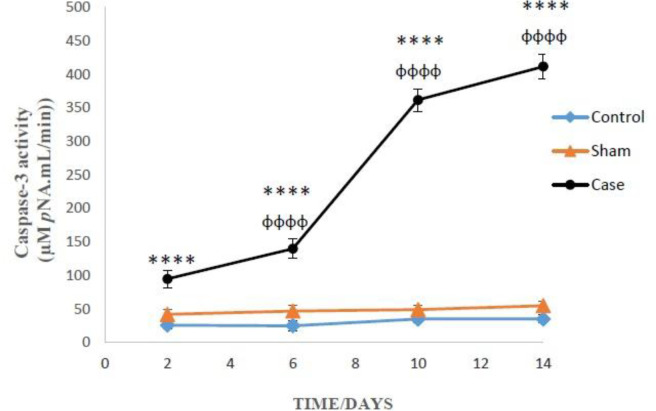
Caspase-3 activity in the glial cells after the induction of neuropathic pain. Values are presented as mean ± SD. ^****^P < 0.0001 Signiﬁcant difference between case and sham groups in corresponding time; ^ɸɸɸɸ^*P* < 0.0001 Compared with previous time in case group

Two-way ANOVA analysis showed significant influence of neuropathic pain on the caspase-3 activity in the glial cells (*P* < 0.0001). However, there was not any significant differences between sham and control groups in caspase-3 activity (Figure 5). Bonferroni *post hoc* tests also indicated that caspase-3 activity in the glial cells of pain group was time-dependent (*P* < 0.0001).

*Effects of neuropathic pain on lysosomal membrane leakiness*

**Figure 6:**
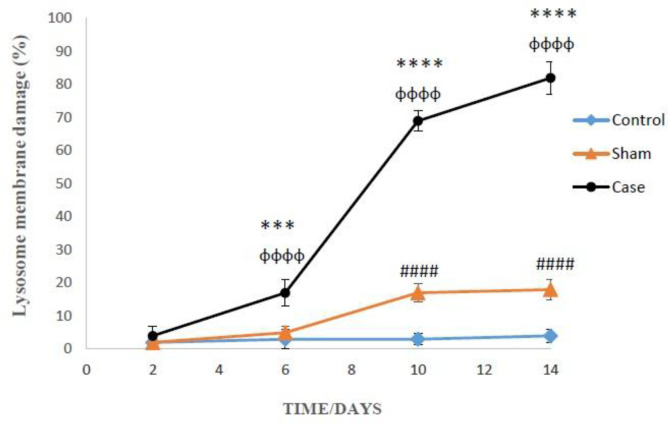
Lysosomal membrane leakiness in the glial cells after the induction of neuropathic pain. Values are presented as mean ± SD. ^***^P < 0.001, ^****^P < 0.0001 Signiﬁcant difference between case and sham groups in corresponding time; ^ɸɸɸɸ^*P* < 0.0001 Compared with previous time in case group; ^#####^*P *< 0.0001 Signiﬁcant difference between sham and control groups in corresponding time

The lysosomal membrane instability in the glial cells of pain groups was significantly elevated in comparison with the sham group in different time points, while the damage was considerably higher in the glial cells of sham group compared to those of control group at 10 and 14 days after the pain induction (*P* < 0.0001) (Figure 6). Again, Bonferroni *post hoc* test indicated that neuropathic pain had time-dependent effect on lysosomal membrane leakiness (*P* < 0.0001).

*Effects of neuropathic pain on cellular proteolysis*

**Figure 7:**
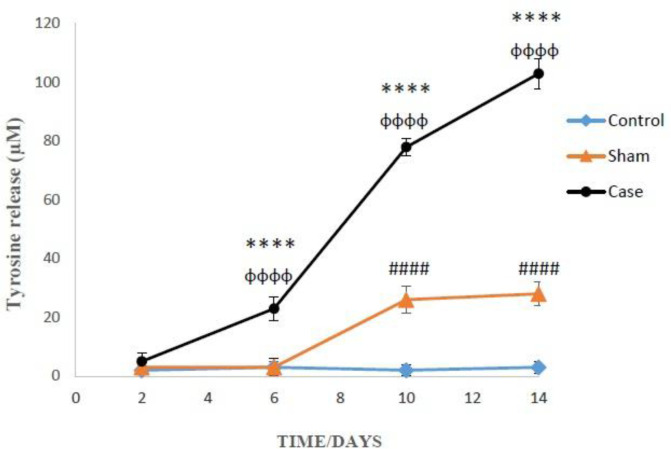
Comparison of cellular proteolysis in the glial cells after the induction of neuropathic pain. Values are presented as mean ± SD. ^****^P < 0.0001 Signiﬁcant difference between case and sham groups in corresponding time; ^ɸɸɸɸ^*P* < 0.0001 Compared with previous time in case group; ^#####^*P *< 0.0001 Signiﬁcant difference between sham and control groups in corresponding time

Finally, the induction of neuropathic pain leads to the release of tyrosine into the extracellular medium of the glial cells (*P* < 0.0001). Moreover, the cellular proteolysis in the glial cells of the sham group was significantly developed compared to the control group at 10 and 14 days after the pain induction (*P* < 0.0001) (Figure 7). Additionally, two-way ANOVA analysis indicated that neuropathic pain had time dependent effect on cellular proteolysis (*P* < 0.0001).

## Discussion

Glial cells include oligodendrocytes, astrocytes, ependymal cells, and microglia are activated during the formation and maintaining of persistent pain induced by either inflammation or the damage to the peripheral tissues and nerves. Upon activation, glial cells release pro-inflammatory mediators, including ROS, nitric oxide, arachidonic acid, leukotrienes, prostaglandins, and cytokines (12). These stimulators can intensify the pain sensation, and it seems that the brain is more sensitive to oxidative stress than other organs (30). Glial cells release proinflammatory cytokines during neuropathic pain that results in the production of ROS more possible from mitochondria. The generated ROS from the affected areas of the brain can consequently target and damage proteins, DNA, lipids, and cellular organelles. Our results indicated that intracellular ROS formation was increased in the glial cells of the rat brain and ROS production reached its highest level in 14 days after the induction of pain.

GSH and the related enzymatic systems belong to the antioxidant defense system protecting the intracellular biomolecules from oxidative damage (31). Inactivation of ROS causes the oxidation of GSH into GSSG that can either be recycled to GSH or removed from the intracellular milieu through specialized transporters. In the present study, the levels of intracellular GSH considerably increased at 2 and 6 days after the pain induction, which could be due to GSH rapid synthesis in response to oxidative stress (32). However, the levels of GSH were rigorously diminished on the 10^th^ and 14^th^ day of pain induction that may be due to severe ROS detoxification in this group. Rapid GSSG formation along with the inadequate levels of GSH indicate the unbalanced redox states and the incidence of oxidative stress, a condition that may lead to cell death. In addition, in this study the role of methionine and betaine as precursors of biosynthesis for GSH was investigated. The obtained results indicated that methionine and betaine as the precursors of GSH biosynthesis triggered GSH synthesis in the affected glial cells yet again. Therefore, the level of GSH in this group was significantly higher than 14^th^ day pain group. Obviously, the reduction of intracellular GSH in pain groups might be solely due to neuropathic pain-induced oxidative stress. 

The reduction of mitochondrial membrane potential is an important indicator for assessing the mitochondrial damage. Thiol oxidation in the mitochondrial membrane by ROS and MPT pore opening have been recurrently used for diagnosis of mitochondrial dysfunction and subsequent cellular events (33). The reduction of mitochondrial membrane potential by MPT pore opening would permeabilize mitochondrial membrane that leads to cellular damage and the initiation of apoptosis. As shown in Figure 4, decline in mitochondrial membrane potential as a marker of mitochondrial damage was increased on the second day of pain induction. It is suggested that the oxidative stress ensued upon the neuropathic pain induction would proceed to the mitochondrial dysfunction. It seems that over the time, the damage to mitochondria going to be more severe and in fourteenth day it was at the highest level.

Our results indicated that caspase-3 activity in the pain group was dramatically increased, which may be due to the cascade of events begin from huge ROS formation after induction of persistent pain. These toxic events eventually proceed to the opening of MPT pore and subsequent releasing of cytochrome C. The release of cytochrome c from mitochondria into cytosol following the MPT pore opening is a key initiating step in both apoptotic and necrotic cell death processes in intact cells (34).

In this study, the effect of neuropathic pain stress on lysosomes was also studied. In recent years, it has been found that partial and selective permeability of lysosomes was followed by the release of proteolytic enzymes especially cathepsins (B, D, L) Apparently, hydroxyl radicals formed through the Haber-Weiss reaction inside the lysosomes (catalyzed by iron), provoke the lysosome membrane damage. Leakage of lysosomal enzymes would increase H_2_O_2_ production in mitochondria and finally lead to further lysosomal permeabilization, since H_2_O_2_ released from mitochondria can easily penetrate into the lysosomes and start the Haber-Weiss reaction again (35). Our results confirmed such cascades of events and show that neuropathic pain could increase lysosomal membrane fragility and leakiness as a consequence of ROS generation that directly contribute to cell death. Lysosomal membrane damage caused cellular proteolysis that was determined by measuring the extracellular tyrosine. It can be said that by promotion of pain duration and intensity, lysosomal leakage and proteolysis occurred in the glial cells of rat brain that ultimately end to cellular death. 

In conclusion, our results conﬁrmed that neuropathic pain has different toxic effects in the rat brain glial cells, including elevated ROS generation, GSH depletion, mitochondrial dysfunction, activation of caspases cascade, lysosomal membrane leakiness, and cellular proteolysis. Our ﬁndings contribute to a better understanding of toxic mechanisms of neuropathic pain in the glial cells of rat brain. 

**Table 1:** Effects of GSH precursors on intracellular GSH in the glial cells on the 14^th^ day after the induction of neuropathic pain

**Groups**	**Intracellular GSH (µM)**			
	**Incubation time**			
	**2 (min)**	**60 (min)**	**120 (min)**	**180 (min)**
Control	67 ± 3	64 ± 4	62 ± 4	60 ± 2
Sham	68 ± 3	58 ± 3	54 ± 4	50 ± 2^##^^#^
Case	22 ± 2^*^^***^	15 ± 2^****^	11 ± 3^****^	6 ± 2^****^
+ Methionine (1 mM)	22 ± 1	32 ± 2^ΦΦΦΦ^	43 ± ^1^^ΦΦΦΦ^	41 ± 2^Φ^
+ Betain (2 mM)	22 ± 2	36 ± 1^ΦΦΦΦ^	43 ± 2^ΦΦΦΦ^	43 ± 3^Φ^

The glial cells were isolated on the 14^th^ day after the induction of neuropathic pain and their intracellular GSH levels were measured during the time. The values are presented as mean ± SD. ^###^*P *< 0.001 Signiﬁcant difference between sham and control groups in corresponding time; ^****^P < 0.0001 Signiﬁcant difference between case and sham groups in corresponding time; ^Φ^*P *< 0.05, ^ΦΦΦΦ^*P *< 0.0001 Signiﬁcant difference between the precursors and case groups in corresponding time.
